# Ketogenic diet and microRNAs: focus on cognitive function

**DOI:** 10.3389/fnut.2025.1545832

**Published:** 2025-02-17

**Authors:** Diana Marisol Abrego-Guandique, Erika Cione, Maria Cristina Caroleo, Diego A. Bonilla, Roberto Cannataro

**Affiliations:** ^1^Department of Health Sciences, University of Magna Graecia Catanzaro, Catanzaro, Italy; ^2^Department of Pharmacy, Health and Nutritional Sciences, University of Calabria, Rende, Italy; ^3^Galascreen Laboratories, University of Calabria, Rende, Italy; ^4^Research Division, Dynamical Business & Science Society – DBSS International SAS, Bogotá, Colombia; ^5^Grupo de Investigación NUTRAL, Facultad de Ciencias de la Nutrición y los Alimentos, Universidad CES, Medellín, Colombia; ^6^Hologenomiks Research Group, Department of Genetics, Physical Anthropology and Animal Physiology, University of the Basque Country (UPV/EHU), Leioa, Spain

**Keywords:** microRNAs, cognitive function, ketogenic diet, ketone bodies, biomarker

## Abstract

Cognition is a mental process of understanding and learning driven by memory. Recent advances in molecular biology and neuroscience have revealed a fascinating interplay between cognitive function and microRNAs (miRNAs). The ketogenic diet (KD) is a low-carbohydrate, high-fat, and adequate-protein diet that triggers the synthesis of ketone bodies, establishing ketosis. Recent and accumulating studies on human and animal models have shown that the KD benefits neurodegenerative diseases, where cognition is affected. The KD can also modulate miRNAs, molecules that are dysregulated in the brains of individuals with Alzheimer’s disease, where cognition is lost. In this mini-review, we provide an overview of the function of miRNAs in neurodevelopment and cognition. We also explore how the KD in human studies can enhance cognitive function and highlight the protective role of microRNAs in neurological conditions.

## Introduction

Cognitive function refers to the mental processes involved in acquiring knowledge, reasoning, and manipulating information. It includes the domains of language abilities, perception, memory, learning, decision-making, and attention (1). Conventional models of human cognition have been hypothesized by cognitive scientists within an information-processing paradigm. The mechanisms underlying cognitive processes remain an active area of research. One growing focus is the role of micronutrients (2, 3) and/or a dietary regimen known as the KD (4) in regulating cognitive function, with microRNAs (miRNAs) emerging as key contributors (5). miRNAs are short, non-coding RNA molecules that play a significant role in post-transcriptional gene regulation in several organs and tissues. They are also used as diagnostic tools in some conditions (6, 7). In addition, Orellana *et al.* proposed miRNAs as potential biomarkers in neurodegenerative disease, identifying four miRNAs with variable expression in patients with Alzheimer’s disease (AD) and frontotemporal dementia (8). The regulatory role of miRNAs has profound implications for cognitive function, as numerous miRNAs are expressed in the brain and actively participate in various neurological processes (9) and cognitive performance (10). In this mini-review, we provide an overview of the role of miRNAs in sustaining neurodevelopment and cognition functions. We also discuss how the KD affects neurological conditions by modulating miRNAs, its potential benefits in AD, and which conserved miRNAs were found to be linked to this condition.

### miRNAs sustain neurodevelopment and cognitive functions

MiRNAs play an important role in synaptic function and neurotransmission (11). The impact of miRNAs on cognitive function begins during the development of cortical neurons, oligodendrocytes, and astroglia (12). These small regulators are essential for the formation and maintenance of neural circuits. miR-124 is the most abundant miRNA in the brain and is known to promote neuronal differentiation and axon growth (13), while miR-9, a neuronal-specific miRNA, is highly expressed in the brain (14), where it controls neural stem cell numbers (15). miR-132 regulates neuronal differentiation and maturation and participates in axon growth, neural migration, and plasticity (16). On the other hand, miR-219 is crucial for the coordinated transition of oligodendrocyte progenitor cells to oligodendrocytes and subsequent myelin formation (17). Although miR-138 expression is also elevated in oligodendrocytes (17), miR-199a-5p and miR-145 play a critical role in their maturation (18).

**Figure 1:**
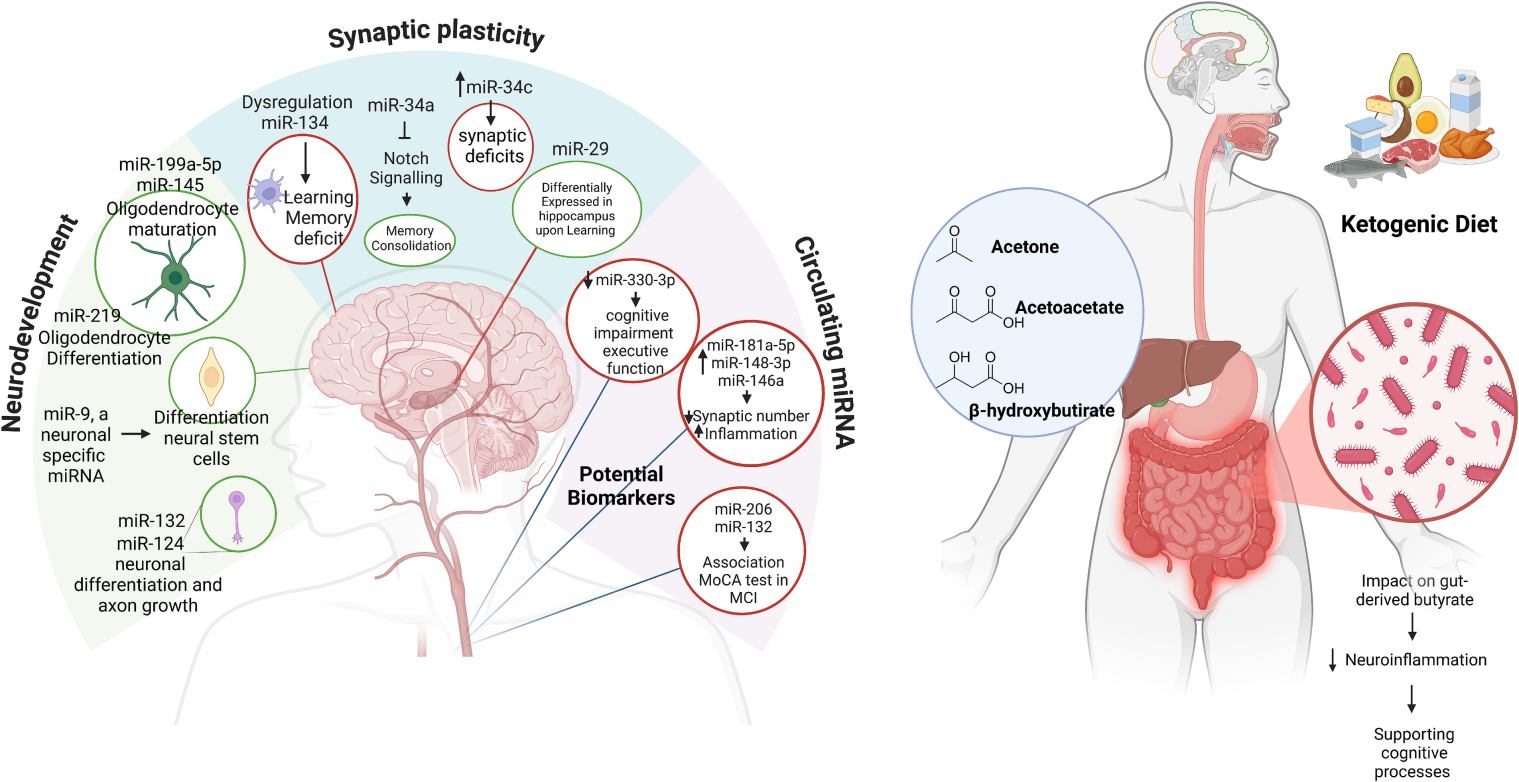
miRNAs in neurodevelopment and cognitive function (on the left). The relationship between miRNAs and their roles in neurodevelopment, synaptic plasticity, and circulating biomarkers in cognitive function and neurological health. Green circles highlight miRNAs associated with positive effects, while red circles represent miRNAs linked to negative effects. MCI, mild cognitive impairment; MoCA, Montreal Cognitive Assessment. The ketogenic diet sustains cognition (on the right). In the blue circle, the ketone bodies produced during the KD; in the dark red circle, the effects of the KD on the microbiome’s synthesis of butyrate. Created in BioRender. Created with BioRender.com.

Synaptic plasticity (SP) is crucial for cognitive function, serving as the foundation for integrated neuronal communication (19). SP is at the core of cognitive function, which includes long-term potentiation and long-term depression, with underlying biochemical mechanisms that support learning and memory (20). miR-134 has been found to play a critical role in modulating SP. For instance, miR-134 inhibits the translation of Lim kinase 1 (LIMK1), which is involved in actin polymerization and dendritic morphology (21). Disruption of miR-134 leads to dysregulation of synaptic plasticity and impaired learning and memory (22). Meanwhile, miR-34a (23) and miR-34c (24) mediate synaptic and memory deficits. The miR-29 family is differentially regulated in the adult hippocampus during learning (25). In addition, miR-466f-3p appears to positively regulate neuronal plasticity by influencing cAMP response element-binding protein (CREB) activity during spatial learning and memory formation in mice (26). Furthermore, studies on miRNAs in brain tissue have shown that circulating miRNAs are also linked to cognitive function (27, 28). miR-212 and miR-484 are essential for synaptic function and neurotransmission (29, 30). Lower expression of miR-484 and miR-197-3p has been associated with accelerated cognitive decline, with downregulation of miR-484 specifically linked to an increased risk of AD development (30). Some studies have found correlations between specific circulating miRNAs and cognitive performance. For instance, Junyi Ma *et al*. found an association between miR-330-3p in serum and executive function (27). MiR-181a-5p, miR-148-3p, and miR-146a-5p showed a significant negative correlation with cognitive function and similar beta-amyloid protein (Aβ) 42/40 ratio values, suggesting their potential as biomarkers for AD (31). In addition, Aβ can inhibit the expression of miR-15a, thereby inducing the expression of Bag5 and activating the protective mechanism of Bag5 against Aβ-induced apoptosis (32). Moreover, miR-206 and miR-132 were positively correlated with the Montreal Cognitive Assessment (MoCA) score in patients with mild cognitive impairment (MCI). Circulating miR-206 and miR-132, which are upregulated in patients with MCI, are proposed as potential biomarkers for MCI diagnosis (28). Notably, of the 17 miRNAs reported here, 11—miR-9, miR-29, miR-34a, miR-132, miR-138, miR-145, miR-148, miRNA-181a-5p, miR-199a-5p, miR-206, and miR-219—are highly conserved across species. This characteristic often enhances the value of basic research from a translational perspective, particularly in AD, where understanding both pathogenesis and treatment-specific health outcomes is crucial (left panel in Figure 1).

### Biochemical pathways in ketogenesis

The biochemistry of ketone body synthesis begins during fasting. Cellular oxaloacetate levels are insufficient to condense with acetyl-CoA for citrate formation, leading to a delay in the citric acid cycle. The essential biochemical process for the KD occurs primarily in the mitochondria of liver cells and follows these biosynthetic steps: (i) condensation of two molecules of acetyl-CoA to form acetoacetyl-CoA; the reaction is catalyzed by the enzyme 3-ketothiolase. (ii) The acetoacetyl-CoA reacts with another acetyl-CoA in the presence of a water molecule, forming 3-hydroxy-3-methylglutaryl-CoA (HMG-CoA) and free CoA, with the reaction catalyzed by HMG-CoA synthase. It is worth noting that this critical step is facilitated by thioester bond hydrolysis, which offsets the unfavorable formation of acetoacetyl-CoA. HMG-CoA lyase cleaves 3-hydroxy-3-methylglutaryl-CoA into acetyl-CoA and acetoacetate, the first ketone body. Acetoacetate, a *β*-ketoacid, has two fates: spontaneous decarboxylation to acetone (the second ketone body) or reduction by NADH-dependent 3-hydroxybutyrate dehydrogenase to form 3-hydroxybutyrate (BHB), the third ketone body (33). Ketosis is elicited not only during fasting but can also occur when minimal amounts of carbohydrates are consumed. For instance, 3 days of consuming 20–30 g of carbohydrates or limiting carbohydrate intake to less than 5% of total daily calories can induce ketosis. The use of this nutritional scheme dates back to over 100 years when it was adopted to manage a form of drug-resistant childhood epilepsy. The neuroprotective effect is considered to be directly due to ketone bodies (34). The KD is a valuable nutrition strategy in the management of recurrent migraines (35). In addition to its antioxidant capabilities, the absence of glycemic peaks during ketosis also causes the production of advanced glycation end products (36, 37).

### Ketogenic diet and its impact on cognitive function: evidence from human studies

Decline in cognitive function is age-dependent and could be a potential target for extending cognitive health span, in contrast to dementia, including AD (38). The prevalence of dementia worldwide affects more than 55 million people, the majority of whom have AD (39). This situation also leads to onerous burdens on society. In developed countries, the U.S. alone has 6.9 million people aged 65 and older living with AD, according to data updated in 2024 (40). Similarly, developing countries account for 5% of the overall prevalence (41). This disorder affects not only the individual patients but also their families and caregivers. Diagnosing dementia is quite challenging as the MCI test may not always provide a clear indication. Clinical trials have suggested that different types of the KD significantly improve the quality of life and daily function and eased AD-related cognitive impairment, especially in individuals without the epsilon 4 allele of the apolipoprotein E gene (APOE ɛ4). This suggests that ketone body metabolism is related to APOE ɛ4 status (37). Individuals harboring APOE ε4 appear to be at higher risk, as recently reported in a meta-analysis conducted in Italy (42), showing a transitional stage between normal brain aging and dementia. Therefore, it is crucial to find effective prevention therapies to delay the onset of disease and slow cognitive decline. Some studies have explained the mechanisms underlying the protective effect of the KD on AD. By increasing BHB, the KD compensates for the brain’s glucose hypometabolism, providing an alternative and efficient energy source (43). In addition, the KD helps clear amyloid-beta (Aβ) plaques, decrease tau hyperphosphorylation, and improve mitochondrial function. Clinical and preclinical studies have demonstrated cognitive improvements and biomarker modulation, highlighting the KD as a potential therapeutic approach for managing AD (44). It also reduces neuroinflammation by suppressing the NLRP3 inflammasome and lowering the levels of pro-inflammatory cytokines such as IL-1β and TNF-*α*. These findings suggest that the KD shows promise in delaying and/or mitigating cognitive decline symptoms sustained by neuroinflammation (45).

### Ketogenic diet and miRNAs

The modulation of miRNAs by dietary regimens, including the ketogenic diet, is well-documented (46, 47). Micronutrients influence miRNA synthesis epigenetically, with recent evidence suggesting a role for exogenous food-derived miRNAs (48). BHB acts as a signaling molecule, regulating miRNA expression and other epigenetic processes (49). The KD modulates miRNAs that regulate genes involved in metabolic and inflammatory pathways, with normalization of antioxidant and anti-inflammatory miRNAs in obese participants post-diet, suggesting an epigenetic role (37, 50). In fact, miR-34a downregulates SIRT1, a hypothalamic NAD + -dependent deacetylase that regulates energy balance, and enhances mitochondrial biogenesis, fatty acid oxidation, and ketogenesis (51, 52). However, studies on miRNAs as predictors of the KD’s therapeutic effects are scarce, with the majority of them focusing on obesity, where circulating miRNA profiles may indicate the risk of complications and monitor weight loss outcomes (53).

### Ketogenic diet and miRNA in neurological conditions

Emerging evidence underscores the complex interplay between miRNAs, the KD, and cognition, with brain-derived neurotrophic factor (BDNF) serving as a pivotal link. BDNF is essential for neurogenesis, synaptogenesis, and memory. It is influenced by miRNAs that regulate its activity and inflammatory pathways (54). The KD promotes neuroprotective effects by modulating miRNA expression (55) and enhancing BDNF function and synaptic activity. Furthermore, the diet’s impact on gut-derived butyrate, which is known to reduce neuroinflammation, highlights its role in supporting cognitive processes (right panel in Figure 1) (56). Recently, the KD, followed for a minimum of 6 months as a treatment for childhood epilepsy, significantly reduced the number of seizures in seven of eight pediatric patients treated with this nutritional regimen. KD-induced modifications in eleven relevant miRNAs were monitored in peripheral blood mononuclear cells (PMBCs). Seven miRNAs were found to be downregulated by the KD in these patients: miR-3978, miR-6726-3p, miR-130a-3p, miR-4758, miR-6745, miR-532, and miR-185-5p. Meanwhile, four were upregulated: miR-4538, miR-602, miR-330-5p, and miR-4673 (57). In a group of patients with pediatric autism, following 4 months of a KD, the levels of miR-134-5p and miR-132-3p were significantly reduced, while the levels of miR-125b-5p remained unchanged and the levels of miR-375-3p increased (56). Finally, our research focused on a 6-week the KD in obese women who self-reported experiencing migraine. We observed a downregulation of miR-211-5p, no changes in brain-enriched miR-382-5p and miR-342-5p, and an upregulation of miR-590-5p, miR-660-3p, miR-34a-5p and, miR-26b-5p (35, 58). Furthermore, it was observed that miRNA-34a, miRNA-132, miRNA-134, and miRNA-330 are commonly associated with neurodevelopment and cognitive functions in animal studies, with miRNA-34a being notably conserved across species.

### Critical considerations of the KD

While the KD shows potential in modulating miRNAs and influencing cognitive and neurological conditions, the findings must be approached with caution. It was reported that low-carbohydrate diet regimens were associated with less confusion and faster response during an attention vigilance task, with a positive impact on cognitive behavior even when followed for 1 year (59, 60). Confounding factors must also be considered, such as individual variability due to genetic polymorphism differences, basal metabolism, and adherence to the diet, all of which significantly influence the outcomes. For instance, genetic polymorphisms in pathways related to lipid metabolism or inflammation may alter the miRNA response to the KD, leading to variable effects on cognitive and neuroprotective outcomes (61). In addition, the KD is associated with potential health risks that must not be overlooked. These include nutrient deficiencies, gastrointestinal disturbances, dyslipidemia, and, in some cases, long-term cardiovascular risks (62, 63). In fact, the low-carb pattern is considered to be more beneficial than very low-carbohydrate diets in terms of cardiovascular mortality (64). These adverse effects highlight the need for personalized approaches when considering the KD as a therapeutic intervention.

## Conclusion

In conclusion, understanding the complex interplay between metabolism during the KD, miRNAs, and cognitive function is important for calibrating the KD regimen to delay neurodegenerative diseases by activating endogenous miRNAs and, consequently, multiple molecular and cellular pathways. Notably, the network of miRNAs and their influence on the KD and cognitive function is a promising area of research in neuroscience to delay the onset of AD.
